# What Are the Causes Which Have Brought about a Change in the General Treatment of Our Diseases, from the Depletive to a Tonic and Stimulating?

**Published:** 1858-07

**Authors:** J. M. Hinkle


ARTICLE VIII.

### WHAT ARE THE CAUSES WHICH HAVE BROUGHT ABOUT A CHANGE IN THE GENERAL TREATMENT OF OUR DISEASES, FROM THE DEPLETIVE TO A TONIC AND STIMULATING?

(Read to the Esculapian Society, May 27th, 1858.

BY J. M. HINKLE, M. D.

I feel myself incompetent to do justice to this subject, and heartily wish that this society had appointed one of our older members to report upon it; one who has seen and observed practice for the last thirty or forty years; such an one, certainly, would have been able to throw more light upon this doubtful subject than I can possibly do. I look on it as a subject of much practical interest to the profession, although it has as yet received but comparatively little consideration. That there exists a necessity for this change in the treatment of our diseases, is a fact which is now generally acknowledged by the profession; that such a change has taken place, is a truth cognizant to every one. But what are the circumstances which have brought about this change is the question.

In setting forth my views on this subject, my opinions may not corroborate and verify the observation of those of you who have witnessed all the causes and circumstances which have brought about this great revolution in medicine; but I offer them as my own conclusions, deduced from all the information which I have been able to gather on this subject, and if I shall only succeed in drawing out the opinions of the members of the society on this subject, I shall not entirely fail of my object.

Before entering into a discussion of the causes which have exerted an influence in bringing about the present prevalent mode of treatment, it may be well briefly to allude to some of the causes which brought the depletive plan of treatment into such general vogue in this country.

There has ever been a disposition in our profession to fall in with the doctrines and theories of some great leading spirit, who, for a time, moulds and gives shape and character to the profession, and who in a great measure dictates and controls the remedial measures used. We find abundant evidence of the truth of this in reading the history of medicine. The oracular teachings which emanated from the temple of Esculapias, and which were afterwards matured and arranged into a system by Hippocrates and his sons, constituted for a long time the most perfect system of medicine known; but these, after a time, were obliged to yield to the invasions of the empirics and methodics, until Galen, by his great powers, wrested medicine from their hands, and revived the old Hippocratic doctrines, and such was his influence over the realms of medicine, that he held undisputed sway for more than twelve centuries; but his doctrines were at length forced to retire before the wild theories of the enthusiastic Paracelsus, who proclaimed to the world that he had discovered a universal panacea, by which immortality might be secured, but who himself died at the age of forty-eight years. Then followed the chemical and humeral doctrine of lentor and thinness of blood, of fermentation, acidity, alkalescency, etc., supported by all the learning, sagacity, and eloquence of the mighty Boerhaave and the renowned Sydenham; but these had in their turn to fall before the spasm and plausible theories of the cautious Cullen, the poetic but ill-authorized notions of Darwin, and the bold and dashing fancies of Brown. This singular genius, Brown, grounded his pathology on the principle that excitability is accumulated during rest and exhausted by exertion, and perpetually renewed for the demands of the system by food and sleep; that health, predisposition and disease depend upon the various relations and proportions of this principle, acted upon by stimulus; that the living state of the body is a forced one, and produced by the action of exciting powers on the excitability. He made all diseases to form but two great classes, those of debility and those of excess of excitement, ninety-seven out of a hundred being of the former class; consequently, the plan of treatment adopted was a tonic and stimulating one. It was during the prevalence of this doctrine that the great apostle of medicine in America, the celebrated Dr. Rush, was receiving his medical education in Edinburgh, and he imbibed to a very considerable extent the doctrines of the Brunonian theory, and by them was influenced in his practice up till 1793, when there broke out in

Philadelphia an epidemic which would not yield to the practised theories of his education. He at once discarded his old notions of pathology, and adopted a depletive plan of treatment, which was attended with marked success. He retained Brown's principle of solidism and excitability, but erased debility from the category of disease. Debility with him was not disease, but only a predisposing cause. He ascribed disease to a morbid action in the living solids, superinduced by the undue application of the exciting powers to the living system. All diseases with him were sthenic or excitement, and the various genera and species of nosologists were merely so many forms of the same disease, for disease with him was a unity; consequently, his therapeutical measures were as simple as his pathology. He contended that after the removal of the exciting causes, to equalize excitement on plans strictly conformable to the state and condition of the system, constituted the most effectual mode of cure; hence the agents used were limited, consisting chiefly of depletives, evacuants and revulsives. Such are some of the sweeping generalities of the revered and renowned Dr. Rush—he who first gave nationality to American medicine, and whose name is indelibly written both in the scientific and political history of our country.

Is it any wonder that doctrines so simple in their nature, so easily comprehended, and yet so plausible, advocated and promulgated by one who had justly acquired the most brilliant reputation of his country, and that too just at the time we were taking our national existence; I repeat it, *is it any wonder* that the great body of American physicians should have adopted the theories and practice of their great master? more especially as there prevailed at that time a sthenic constitution, which strongly verified the popular theory, and lent a powerful influence in establishing the depletive practice.

The enthusiastic young physician, filled with idea of the unity of disease, that all the morbid conditions of the system consisted in an excess of excitement, and inspired with all that confidence in his own opinions, and in the certainty of medicine, which was peculiar to *Rush*, embarked in the field of practice, armed with his "lancet and his ten and ten," indiscriminately depleted every case he met, it mattered but little what the indications were. The consequences of such an exclusive plan of treatment were injurious and derogatory to the profession, for our diseases gradually assumed a more asthenic character, but this change was not at first accompanied by a corresponding change in treatment, but the depletive plan was continued with unabated energy. The consequence was that these abuses opened wide the field for quackery; the most ignorant and presumptuous quacks took advantage of them, and built for themselves an ephemeral reputation upon the excesses into which the regular profession had run; and I verily believe, could they have had science to back them, they would have established upon a more permanent basis; but they were men devoid both of principles and the rudiments of an education, they had no knowledge whatever of the organization of the system upon which they practiced, and their system too was exclusive and in many things absurd; consequently, they committed the most gross and culpable errors, and soon deservedly fell into disrepute. Yet these short-lived quacks, whose influence at one time threatened to overrun the whole country, were not without their benefit to the regular profession. They caused the profession to think, and to observe more caution in the use of the remedies dictated by their old and preconceived notions, and in this way to materially modify both their theory and practice.

The present advanced condition of our pathological knowledge has contributed a considerable influence in bringing about the change under consideration. In the beginning of the present century, pathology was almost entirely conjectural; the means afforded for gaining a correct pathological knowledge were limited; chemistry had as yet thrown but little light upon this subject—it had not made known the elementary constituents and definite proportions of the blood and other vital fluids, nor had the microscope poured forth its revelations of minute and elementary structure, or shed its flood of light upon the changes which take place in pathological lesion.

The science of physical signs as yet had contributed no influence in solving the problem of disease. Many of our most valuable remedies were unknown to the profession. By the aid of all these, pathology has been developed and is still developing into a science. Many diseases, the pathology of which a few years ago were entirely conjectural, are now known and understood by the profession, and the treatment directed to them is upon more rational grounds; for example, scarlatina, which, in the beginning of the present century, was treated as a high inflammatory grade of fever, but now it is known to be one of disturbed constituency of the blood, the constant tendency of which is to putrescency.

How far what is generally termed a change of disease, or of the prevailing diathesis which characterizes our period, has had to do in bringing about a corresponding change in treatment, I can hardly say; but that it has exerted a potent influence I do not doubt. I have conversed with a number of physicians on this subject, some of whom have been practising for the last thirty or forty years,—they give in almost their united testimony that for the last fifteen or twenty years there has been a tendency in all our diseases to assume a typhoid form, that mucous inflammations have prevailed rather than serous ones, that neuralgias and nervous complaints have been more common, and that hepatic and bilious complaints have been less frequent. Dr. Drake predicted many years ago that such would be the case. He grounded his opinion upon the changes in the manners, customs and habits of the people, and upon the influence which drainage and cultivation would have on our soil. But to my mind this does not sufficiently explain the problem. I cannot see that the limited change which has taken place in the manners and customs of our people, or even the partial change in our soil, could have such a general and universal effect over our whole country. Nor do I endorse the theory of Dr. Read, who incidentally said, in a paper read before us at our last meeting, that the reason why nervous diseases and diseases of debility now predominated, was, because of the continued state of excitement and high nervous development which had characterized the American people for the last quarter of a century. He said, in this time of railroads and telegraphs, of steamboat explosions and terrible conflagrations, and a thousand other circumstances calculated to fill the mind with anxiety and emotion, the nervous system had been kept upon a continued stretch of excitement, until the American people have become completely enervated, and subject to a train of diseases which follow upon such a state of the constitution.

If such causes would bring about such an effect, then surely after the long and exciting struggle of the revolution, we would have had in America a period conspicuously marked by the asthenic character of the diseases which then prevailed, but history tells us that such was not the case; but there existed at that time an opposite state of constitution, and I know not that startling intelligence, either good or bad, conveyed on Morse's electric telegraph, would have a different effect upon the human economy from the same conveyed on our old hackney coaches.

And the masses of the people inhabiting our country live remote from these influences, and neither know nor care anything about them, consequently, they could not in any material degree be effected by them. I have not as yet found any satisfactory reason which will account for this much talked of reigning typhoid element, which enters into the composition of all our diseases. We can only say that it is epidemic influence, but what that epidemic influence is, is left in a great measure to hypothesis.

I have thus briefly hinted at some of the causes which I believe to have exerted an influence in changing the general plan of treatment adopted by the profession from the depletive to the tonic and stimulating; these causes, all acting in concert, were powerfully assisted by the results of experience. The almost unanimous testimony of the profession is, that as this change gradually took place, the increased success attending it fully justified it. Practitioners found by experience that their patients bore less and less the free use of the lancet, and required more and more the tonic and bracing treatment. In this way the change almost imperceptibly progressed, until it has amounted almost to a complete revolution in medicine. But before leaving the subject, I will speak of one other cause which I have not yet referred to; that is, the influence which the popular theories and practice of the day has in dictating and controlling our practice, and shall also allude to the evident tendency which now exists in our profession to adopt exclusively the tonic and stimulating treatment, and shall try to call the attention of this society to the great danger which will inevitably follow upon such a course.

A witty writer has said—

> "Fashion in everything bears sovereign sway,
> And words and periwigs have each their day."

This is not only true of the passing frivolities of the day, but it is equally applicable to the weightier matters of the law and gospel, and even to a profession like ours, which should alone acknowledge the influence of stern reason, accurate research, and legitimate deduction. That fashion has very much to do in influencing our practice, is a truth which we have all felt and must acknowledge, especially in the use of one article in our materia medica, which has been introduced into popular use within the last thirty years. I refer to quinine, which, from its peculiar adaptation to the treatment of our malarious forms of disease, has become an essential part of almost every prescription, and is now almost as indiscriminately used as the lancet and calomel were forty years ago. It is now recommended in almost every form of disease which occurs in our country, from the slightest functional derangement, to the gravest form of disease; in proof of which, I only need refer you to the popular journals of the day.

That it is a valuable remedy, and that it possesses as great if not greater remedial powers over the diseases of our country than any other one remedy, I do not deny, and the experience of the profession will bear me out in this. But to make it as some almost do, the *sine qua non*, the universal remedy, before which disease, in all its hideous forms, takes flight and leaves the fertile plains of the Wabash Valley, is taking grounds which I cannot occupy. This, I admit, is a little extravagant, but we have had such a doctrine, slightly modified, advocated with ability in this society since I have been a member. Cases of prolapsus uteri have disappeared under the use of quinine, and pneumonia no longer possesses any terrors, for its most alarming attacks can certainly be cut short in from twelve to twenty-four hours, by this all-powerful remedy.

Now I ask you gentlemen, does your experience verify this theory? have you seen such magical results from the use of quinine in such cases? I confess that I have not. And where is the practitioner of medicine in this country who has not seen and felt convinced of the often unnecessary, if not positively injurious use of this most valuable remedy; and who among us has not prescribed it in cases where his judgment has dictated to him a contrary course? But such is the irresistible influence of fashion, that we are almost involuntarily carried into this channel of popular practice.

It is time the profession were waking up on this subject, for inattention to it may prove to be dangerous.

If we have seen the evils flowing from an exclusive adherence to any of the various systems which have characterized the different periods of medical history, let us profit by such knowledge, and guard well against falling into a like error. If the extreme into which the profession run in the use of depletive remedies was made a means of bringing a reproach upon it, may we not expect a like result if we run into the opposite extreme, and I verily believe that there now exists an evident tendency in the profession to occupy that ground. If we thus incautiously permit ourselves to fall into abuses and errors, and fail to correct them, quacks will do it for us, and our noble and exalted profession will be made to suffer for faults in us which we might have prevented.

